# 2026 10^th^ International Conference on Medical and Health Informatics (ICMHI 2026)Japan, 15 – 17^TH^ May 2026

**DOI:** 10.1515/med-2026-1526

**Published:** 2026-10-02

**Conference President**

Prof. Chi-Chang Chang

**Scientific Committee**

Prof. Taka-Aki Sato

Prof. Tetsuya Sakurai

Prof. Wen-Wei Chang

Prof. Ahmet Murat Özbayoğlu

Prof. Yen-Wei Chu

Prof. Chi-Jie Lu

Prof. Chih-Te Yang

Prof. Hao-Yun Kao

Prof. Chien-Lung Chan

**Organizing Committee**

Prof. Alvin Marcelo

Dr. Xiucai Ye

Prof. Rung Ching Chen

Dr. Bolormaa Purevdorj

Prof. Tian-Fu Lee

Dr. Kittipat Charoenkwan

Prof. Ahmet ALTUN

Dr. Roberto Gatta

Dr. Hsien-Wei Ting

## Designing an Equitable EHR-Integrated Medication Management Tool

Sreyon Murthi^1^, Nataly Martini^1^, Shane Scahill^1^, Nazanin Ghahreman-Falconer^2^

^1^University of Auckland, New Zealand

^2^University of Queensland, Australia

Medication-related harm is a leading cause of preventable morbidity and mortality in hospitals. Electronic health records (EHRs) and electronic medication management systems (eMMS) aim to improve safety through standardisation and clinical decision support, yet usability limitations, fragmented workflows, and insufficient attention to equity compromise their effectiveness, particularly for marginalised populations including Indigenous peoples, ethnic minorities, older adults, and those with communication or cognitive barriers. To identify barriers, equity gaps, and design priorities for safer, more culturally responsive, and equitable eMMS. Methods: This participatory qualitative study took place at a major tertiary hospital in Auckland, New Zealand, during 2025. Twenty-two staff from pharmacy, nursing, medicine, digital health, and leadership took part across 13 semi-structured interviews and two focus groups. Transcripts underwent reflexive thematic analysis (Braun and Clarke), interpreted through four lenses: User-Centred Design, the Sociotechnical Model, the Digital Health Equity Framework, and the four-pillar Māori health model Te Whare Tapa Whā. Methodological rigour drew on reflexive journaling, supervisory oversight, and COREQ-guided reporting. Three interconnected themes emerged. First, design and organisational factors undermined safety, as cluttered interfaces, inflexible automation, and unstable systems raised mental workload and forced sustained manual oversight. Second, systems obscured cultural and social context; without structured fields for language, disability, and family involvement, culturally safe communication and equitable care were constrained. Third, participants prioritised safer, more inclusive systems: responsive decision support, consolidated patient views, and built-inequity features such as interpreter prompts, disability and family fields, and continuing co-design. Safer medication use depends on more than digitisation. Digital tools must develop in step with clinical, social, and cultural practice, with usability, reliability, and equity treated as core safety functions. These practitioner-derived findings give a concrete basis for co-designing EHR-integrated medication systems that areresponsive to context, culturally grounded, and clinically useful.

## The Feasibility and Effectiveness of Artificial Intelligence in Addressing Language Barriers in Healthcare Settings: A Systematic Review

Quoc Dung Nguyen^1^, Dipankar Srirag^1^, Joseph Zhu^1^, Roberto Forero^1^, Ben Harris-Roxas^1^, Padmanesan Narasimhan^1^

^1^The University of New South Wales, Australia

Language barriers in healthcare contribute to disparities in patient safety and outcomes. While professional interpreters are the gold standard, their use is limited. Artificial intelligence (AI) offers a scalable solution, but its real-world feasibility and effectiveness remain unclear. We conducted a systematic review following PRISMA 2020 guidelines, searching MEDLINE, EMBASE, CINAHL, Scopus, IEEE Xplore, and ACL Anthology from inception to August 2025. We included studies evaluating AI tools (e.g., LLMs, machine translation, chatbots) for language translation or communication in healthcare settings. From 12,562 identified records, 22 studies were included. AI tools were found to be technically feasible and acceptable for low-risk tasks (e.g., translating standard instructions, simplifying educational materials). However, effectiveness was highly language dependent. Performance was robust for high-resource languages (e.g., Spanish) but dropped significantly for low-resource languages (e.g., Haitian Creole, Urdu, Somali), leading to frequent clinically significant errors. AI underperformed human interpreters in nuanced communication. Key barriers included workflow integration challenges, data privacy concerns, and a substantial risk of exacerbating health inequities for speakers of minority languages. AI presents a promising but nascent tool for addressing healthcare language barriers. Current evidence does not support its use as a replacement for professional human interpreters. Instead, AI should be cautiously integrated as a supplement for specific, low-risk tasks, with mandatory human oversight, rigorous validation for local languages, and strong governance to ensure safety and equity.

## Radiomics-Based Detection of Coronary Artery Calcium on Scout Images for Opportunistic CAD Screening

Yu-Tai Shih^1 2^, Jia-Chi Hsu^3^, Jay Wu^3^

^1^Hualien Tzu Chi Hospital, Taiwan

^2^Tzu Chi University, Taiwan

^3^National Yang Ming Chiao Tung University, Taiwan

Coronary artery disease (CAD) is closely linked to metabolic syndrome and remains a major cause of mortality worldwide. Coronary artery calcium (CAC) is typically assessed using cardiac CT with the coronary artery calcium score (CACS), but the cost and radiation dose limit CT-based screening. This study proposes a radiography-based coronary artery calcium system (RACS) using radiomics to enable opportunistic CAD risk assessment. We retrospectively analyzed lateral scout images (LAT) from cardiac CT in 300 patients with known CACS. The cardiac lateral view was manually segmented, and 184 radiomic features were extracted. Feature selection included Kruskal–Wallis testing, Spearman correlation filtering, and LASSO L1 regularization, yielding five core features. Random Forest (RF) and Support Vector Regression (SVR) models were developed for continuous CAC prediction and binary classification at CAC thresholds >50, 100, 200, and 400. The RF model outperformed the SVR model in all tasks. RF demonstrated strong correlation with CACS (R^2^ = 0.839), while SVR performed poorly (R^2^ = 0.292). For binary classification, RF achieved high discrimination, with AUCs increasing at higher thresholds (0.886 for CAC >100; 0.961 for CAC >400). Overall RF performance metrics included MAE 171.5, RMSE 279.6, and F1-score 0.832. Feature-importance analysis indicated that GLRLM features describing high-density continuity were key predictors. Radiomics analysis of cardiac lateral images can effectively estimate CAC burden and support opportunistic CAD screening. The proposed RACS approach demonstrates accuracy, robustness, and strong potential as a noninvasive risk-stratification tool. This approach may be extended to routine lateral chest radiography in future clinical practice.

## Predicting future suicidal ideation and identifying persistent risk and protective factors among college students through multiple machine learning techniques: A two-year follow-up longitudinal study

Li-Chung Lin^1^, Chih-Han Leng^2^, Grace Yao^3^

^1^Chung Yuan University, Taiwan

^2^National Tsing Hua University, Taiwan

^3^National Taiwan University, Taiwan

University students exhibit higher rates of suicidal thoughts and behaviors than the general population, particularly in Asia. Although machine learning has been increasingly used to predict suicide risk, prior studies have rarely incorporated longitudinal data or simultaneously examined the effects of protective, risk, and interactive factors in predicting persistent suicidal thoughts and behaviors. This longitudinal study developed a screening algorithm for persistent suicidal thoughts and behaviors among university students and identified key protective and risk predictors using longitudinal data from 1,199 students at National Taiwan University collected in 2023 and 2024. Sixteen standardized measures assessed suicide-related outcomes, demographic characteristics, mental health, and protective and risk factors. The Synthetic Minority Over-sampling Technique (SMOTE) was applied to address class imbalance. Five predictive models—logistic regression, bagged logistic regression, random forest, LightGBM, and support vector machines—were developed and evaluated using the 2023 dataset. The best-performing model was then used to predict suicidal thoughts and behaviors in 2024 based on participants’ 2023 responses, and its feature importance was subsequently examined. Machine learning demonstrated strong utility for suicide risk prediction among university students. Among the models evaluated, random forest achieved the best performance (Accuracy = 0.8624, Specificity = 0.9003), followed by support vector machines with an RBF kernel (Accuracy = 0.8499, Specificity = 0.8923). The most influential predictors included emotional instability and psychological pain resulting from life changes, enjoyment of family interactions, unexplained fear and anxiety, and perceived family satisfaction with one’s academic pursuits and goals. These findings highlight key psychological and family-related factors associated with persistent suicide risk. The proposed machine learning-based screening system may improve early risk identification and support school-based psychologists and counselors in implementing timely suicide prevention interventions.

## Predictive Scoring Model and Indicators of Intestinal Parasitic Infections in School-age Students: A Cross-sectional Study in Jalalabad City, Afghanistan

Ahmad Gul Azami^1^, Sant Maungnoicharoen^2^, Polrat Wilairatana^2^, Noppadon Tangpukdee^2^

^1^Nangarhar University, Afghanistan

^2^Mahidol University, Thailand

Intestinal parasitic infections (IPIs) remain a global health concern and continue to be an important public health problem in developing countries, particularly in low-income settings. School-age students (SAS) are particularly vulnerable subjects due to lack of appropriate personal hygiene and poor socioeconomic conditions. The diseases may cause them serious effects and can negatively impact their health status and cognitive developments. Currently, there is limited data of IPIs in SAS living in the city of Afghanistan. This study aimed to devise a reliable predictive model and investigate the indicators associated with IPIs in SAS lived in Jalalabad, Afghanistan. The cross-sectional, laboratory-questionnaire-based study was conducted in accordance with the ethical principle outlined in the Declaration of Helsinki. 603 students from 8 randomized schools in Jalalabad were recruited. Written or thumbprint informed consents were obtained from legal guardians of students. Stool samples were examined using a formal-ether concentration method. Potential indicators of IPIs were identified by univariable and stepwise multiple discriminant analyses. Receiver Operating Characteristics (ROC) curve was applied for determining the performance of the model. The prevalence of IPIs was 48.8%. The predictive model (Y) = 1.23(older age group) + 0.91(always eating street food) + 1.55(only sometimes handwashing after using latrine) + 1.98(poor handwashing before eating) + 3.29(never washing hand after arriving home) + 3.29(always eating unwashed raw fruits and vegetables + 1.68(poor nail hygiene) – 3.93. The area under the curve of ROC was 90.0% (95%CI: 87.6–92.5) with a sensitivity, specificity and accuracy of 86.7%, 75.1% and 81.1%, respectively. Almost half of SAS in Jalalabad were infected with IPIs, implying a significant public health concern. Public health informatics for policy and management should be urgently targeted with personal hygiene and school-community health education to alleviate the burden of IPIs in targeting population and community all around.

## AI-Assisted Ultra-Processed Food Identification: Evaluating Large Language Models for NOVA Classification of Taiwanese Foods

Hsiao-Wen Chuang^1^, Yi-Chen Huang^1^

^1^China Medical University, Taiwan

The NOVA classification system categorizes foods into four groups based on their degree of processing and has become increasingly important in nutritional epidemiology. However, existing literature has predominantly focused on Western dietary patterns and primarily relies on machine learning algorithms or self-developed systems. To date, no study has examined image- and text-based NOVA classification for Asian composite dishes. This study aimed to evaluate the feasibility of AI-assisted food processing classification for dietitians. This cross-sectional study selected 30 common dishes and 133 food ingredients from the 2013–2016 Nutrition and Health Survey in Taiwan (NAHSIT). We compared the accuracy of NOVA classification across three AI models (GPT-5, Gemini, Claude Sonnet 4) and 32 human evaluators (11 clinical dietitians, 11 nutrition undergraduates, and 10 individuals from the public). Chi-square tests with Bonferroni correction were used for statistical analysis (α=0.05). For dishes, AI achieved a mean accuracy of 82.2%, significantly higher than humans (55.2%; p<0.001). For ingredients, AI accuracy was 71.2% versus 66.6% for humans (p=0.074). AI demonstrated particular superiority in identifying ultra-processed foods (NOVA 4), with accuracy of 66.7% vs 40.6% for ingredients (p<0.001) and 80.0% vs 47.5% for dishes (p=0.001). Claude showed the highest accuracy for ultra-processed food ingredients (77.3%). Humans outperformed AI only in classifying culinary ingredients (76.1% vs. 64.8%, p=0.013). Large language models outperformed humans in NOVA classification, particularly in ultra-processed foods. A human-AI collaborative approach is recommended, where LLMs provide initial classifications followed by professional verification from dietitians, balancing both efficiency and accuracy.

## Expanding Access to Pediatric Clinical Services in Ukraine’s Conflict Zones Through Telehealth

Stella Nam^1 2^, Mary Showstark^1^, Yuliia Kardash^1^, Oleksii Vlasov^1^, Olha Maltseva^1^, Robert Arnot^1^, Karen Redlener^3^, Irwin Redlener^3^, Jarone Lee^1 2^

^1^Health Tech Without Borders Inc., Boston, USA

^2^Massachusetts General Hospital, Boston, USA

^3^Ukraine Children’s Action Project, New York City, USA

As of February 2025, an estimated 2.9 million children in Ukraine are in urgent need of humanitarian assistance, particularly improved healthcare access. The ongoing conflict and disrupted power infrastructure have heightened health risks. In response, Health Tech Without Borders (HTWB) and the Ukraine Children’s Action Project (UCAP) partnered together to expand quality care for children with complex needs through a telehealth program. The goal was to implement a telehealth program to improve access, quality, and efficiency of pediatric care across four hospitals in Dnipro, Ukraine. We conducted a needs assessment of clinical workflows and telehealth infrastructure, evaluated platforms, optimized workflows, and supported procurement and training for mobile imaging and video teleconsultation technologies. A pilot launched in June 2024 tested solutions and workflows, leading to a full launch in May 2025 focused on sustainable telehealth. Throughout, we monitored impact and facilitated training with international pediatric experts for complex cases. From May through October 2025, the telehealth program facilitated 783 consultations involving 407 patients (69.5% pediatric, 30.5% adults). The top pediatric specialties were palliative care (20.1%), psychiatry (12.4%), and trauma (8.5%), while among adults the leading specialties were palliative care and endocrinology (14.5%), followed by dermatology (10.5%). The program has expanded beyond four hospitals to include direct home-based consultations, which increased at an average monthly growth rate of 124%. The system also supports cross-border consultations, with Dnipro clinicians providing care to Ukrainian patients abroad and receiving expert input from international partners, including Massachusetts General Hospital in Boston and healthcare providers in Turkey. The telehealth program in Dnipro has shown early success in expanding care access for vulnerable populations, especially children, in conflict-affected Ukraine. These results highlight a scalable, adaptable digital health system ready to support urgent and long-term healthcare needs locally and beyond.

## Using Public Health Informatics and National Claims Data to Identify Inappropriate Cancer Screening in Taiwan

Ying-Chun Li^1^, Hsin-Ching Yang^1^, Pei-Jane Wu^1^

^1^National Sun Yat-sen University, Taiwan

Inappropriate cancer screening places unnecessary burdens on publicly funded healthcare systems and may weaken the effectiveness of preventive service programs. Nevertheless, few studies applied big data analytics regarding this issue in Taiwan. This study utilizes Taiwan’s National Health Insurance (NHI) claims database, which is publicly accessible, along with health informatics and big data analytics to assess the prevalence, determinants, and financial impact of guideline-discordant screening practices. Three types of potentially inappropriate screenings were examined: colorectal cancer screening among adults aged ≥75, cervical cancer screening for women aged ≥65, and PSA testing for men aged ≥75. Study results showed inappropriate screening rates of 6.16% for colorectal cancer, 11.71% for cervical cancer, and 30.08% for prostate cancer. Prostate cancer screening had the highest level of inappropriate use, while colorectal cancer screening incurred the highest average medical costs. Multivariate logistic regression and generalized linear models indicated that demographic factors such as age, gender, income level, and rurality, along with healthcare facility types, significantly predicted the likelihood and costs of inappropriate screening (p < 0.01). This study demonstrates how population health informatics can be used to monitor preventive service quality, identify systemic inefficiencies, and support data-driven policy planning. Combining evidence-based guidelines with informatics-based surveillance can enhance resource allocation, reduce unwarranted variations, and promote equity in Taiwan’s national cancer screening programs.

## Addressing the Urgency of Undiagnosed Ventricular Septal Defects: A Deep Learning Approach for Early Detection in Resource-Limited Settings

Taufiq Hidayat^1^, Irwanto Irwanto^1^, Shabrina Imanina^1^, Nur Iriawan^2^

^1^Airlangga University, Indonesia

^2^Institut Teknologi Sepuluh Nopember, Indonesia

Ventricular Septal Defect (VSD) is the most common congenital heart defect and can lead to severe complications and increased mortality if not detected early. In developing countries such as Indonesia, limited access to pediatric cardiologists contributes to delayed diagnosis and treatment. This study evaluated the performance of YOLOv4-based deep learning models for automated VSD, detect VSDs from ultrasound videos to support early diagnosis. A total of 25 annotated pediatric echocardiography videos from a tertiary hospital in Indonesia were used. YOLOv4 and YOLOv4-tiny models were trained to detect and localize VSD using bounding box annotations. The dataset was divided into 80% for training and 20% for testing. Model performance was evaluated using mean Average Precision (mAP), precision, recall, and detection speed as metrics. Detection results were further validated by expert pediatric cardiologists. The YOLOv4 model achieved high accuracy, with a training mAP of 98.36% and a testing mAP of 95.56%, along with precision and recall exceeding 99%. YOLOv4-tiny, achieved a slightly lower testing mAP (87.24%) while providing superior detection speed at 132 frames per second (fps), making it suitable for real-time applications. Both models successfully identified subtle and complex VSD presentations, aligning closely with expert evaluations. These findings demonstrate the feasibility of deploying AI-driven tools to enhance diagnostic accuracy and efficiency in resourceconstrained environments. This study showcases the potential of YOLOv4-based deep learning models to provide an accessible, efficient, and accurate diagnostic aid for pediatric cardiologists and general practitioners. By bridging gaps in specialized care, particularly in underserved and rural areas, this approach can significantly improve early diagnosis and timely management of VSD. Future research should extend this work to include other congenital heart defects and explore its integration into broader healthcare frameworks to improve outcomes for pediatric patients worldwide.

## Bridging the Implementation-Reality Gap: A Clinician-Led No-Code Platform to Optimize Anesthesia Handovers and Workforce Management

Ratikorn Boonchai^1^

^1^Prince of Songkhla University, Thailand

Spatial fragmentation in complex tertiary healthcare systems often creates information silos, causing rounding fatigue and communication delays. Managing 40 operating theaters across three buildings and 8–10 scattered procedural rooms at a Southern Thailand institution placed a high workload on leadership. This study evaluates a clinician-led intervention designed to decouple situational awareness from physical presence using a digital common operating picture. Utilizing an agile methodology and the Glide low-code/no-code (LCNC) platform, an anesthesiologist developed a mobile-first shadow system within a 60-hour cycle. The application digitized the I-PASS framework, incorporating a comprehensive personnel directory, real-time theater statuses, card-based clinical summaries, and an overtime (OT) self-registration portal. A sequential mixed-methods design (N=114) evaluated the system via the System Usability Scale (SUS), Technology Acceptance Model (TAM), and longitudinal comparison of workflow metrics. The intervention achieved a 94.55% data capture rate over five months. Implementation significantly reduced median rounding time by 55.6% (45 to 20 minutes, p < 0.001) and communication frequency by 50.0% (10 to 5 calls/shift, p < 0.001). The system earned an “excellent” usability rating (mean SUS: 83.4 ± 14.6), with high TAM scores (≥4.31/5.0) for usefulness, workforce efficiency, decision speed, and handover effectiveness. Qualitatively, 77.2% of users reported the app successfully substituted physical rounding. Key drivers of reduced communication latency included real-time theater visibility, the granularity of intraoperative data within the I-PASS modules, and the streamlined self-registration OT portal. Conclusion This study illustrates that clinician-led no-code solutions can effectively bridge the interoperability gaps inherent in traditional top-down HIS models. By transforming mental checklists into a digital twin of the surgical suite, we have converted administrative latency into proactive clinical oversight. This democratization of software development presents a scalable and cost-effective model for enhancing clinical agility and workforce sustainability in high-acuity medical settings.

## Time-Resolved Oximetry-Driven Obstructive Sleep Apnea Event Mapping: A Multimodal Framework with Uncertainty Quantification

Shinn-Ying Ho^1^, Wei-An Ke^1^, Yen-Tze Liu^1^

^1^National Yang Ming Chiao Tung University, Taiwan

^1^Changhua Christian Hospital, Taiwan

The apnea–hypopnea index (AHI) compresses nocturnal respiratory dynamics into a single average value, discarding temporal structure and event clustering that may contain informative signals. This aggregation bias limits the ability of learning-based systems to model structured nocturnal dynamics from wearable data. We propose a time-resolved, oximetry-driven multimodal learning framework that predicts apnea–hypopnea event counts over sliding 2-minute windows, reframing sleep apnea assessment as a temporal regression problem. The model integrates wrist-worn oximetry signals (SpO_2_, pulse rate, respiratory surrogates, and motion), questionnaire-derived risk indicators, and routine health variables. To address feature redundancy and cross-modal heterogeneity, the framework employs evolutionary learning with an intelligent genetic algorithm for data-driven feature selection, jointly optimizing predictive performance and model compactness. To support reliable decision-making, we further incorporate uncertainty quantification via Monte Carlo Dropout with post hoc calibration, producing predictive intervals and case-level confidence scores. The model was trained and internally validated on 178 polysomnography-labeled subjects. It achieved moderate-to-good agreement for nightly AHI estimation (ICC = 0.70, Pearson r = 0.80, MAE = 11.33 events/h) and strong discriminative performance for severe obstructive sleep apnea (AHI ≥ 30; AUC = 0.97). Window-level event prediction indicated learnable temporal structure from wearable signals. Feature importance analysis highlighted contributions from anthropometric measures, oxygen desaturation indices (ODI3%), and SpO_2_ variability metrics. These results demonstrate that temporal modeling with uncertainty-aware learning can recover structured nocturnal dynamics from wearable oximetry, offering a principled alternative to coarse aggregation and suggesting broader applicability to time-series health modeling under weak supervision.

## Predicting the location of death for hospice shared care patients using the random forest algorithm

Chieh-Kuan Hsu^1^

^1^Cathay General Hospital, Taiwan

Passing away at home rather than in a hospital is often considered a good death for end-of-life patients. Early prediction of the location of death allows for timely allocation and arrangement of medical resources. This study aims to use supervised machine learning algorithms to establish a preliminary predictive model to identify high-risk groups for in-hospital mortality. This study retrospectively analyzed 1,409 end-of-life patients aged 18 and above who received hospice shared care (HSC) services at a medical center between 2012 and 2024 and whose case was closed due to death. Forty-nine characteristics were selected, including demographics, disease status, physical symptoms, psychological status, spiritual status, hospice care measures, and medical resource utilization. “Hospital death (Yes/No)” was the binary target variable. This study used Python to train a random forest classifier. Hyperparameters were optimized using grid search with stratified 5-fold cross-validation (5FCV). Class weights and Youden’s J statistic were applied to determine the optimal prediction threshold. Finally, 5FCV on the full dataset was conducted to assess model stability. The random forest model exhibits good discriminative ability, with test set results showing an area under the ROC curve (AUC) of 0.95 (mean AUC of 0.91 ± 0.01 after 5FCV). The optimal decision threshold is 0.49. For the target variable “death in a hospital,” the model’s predictions are: Precision = 0.98, Recall = 0.93, F1-score = 0.95, and overall Accuracy = 0.91. Feature importance analysis shows that “hospitalization within 30 days before death,” “emergency department visit within 30 days before death,” and “Hospital stay longer than 14 days” are the three most important features affecting the prediction results. This study established a preliminary predictive model with good stability and generalizability, representing a pioneering contribution to the identification of high-risk patients who die during hospitalization.

## Development of a risk discrimination model for delirium in non-induced patients in the surgical intensive care unit based on machine learning methods

Yu-Lu Huang^1^

^1^Cathay General Hospital, Taiwan

Delirium is a common ICU syndrome, and early prediction allows timely intervention and prevention of complications. The E-PRE-DELIRIC model uses logistic regression with nine admission indicators to estimate delirium risk, but mainly relies on known high-risk factors and may be less effective in non-induced patients without medication effects, psychiatric disorders, or organic brain lesions. This study developed a rapid model to predict delirium risk in such non-induced ICU patients. This study used Python to develop a logistic regression model using data from 934 ICU patients aged ≥20 years at a northern Taiwan medical center. After excluding patients with heavy sedative use, psychiatric disorders, or organic brain lesions, 17 delirium-related variables were evaluated, with delirium as the binary outcome. Forward stepwise selection based on likelihood ratio tests identified three key predictors. Grid search and 5-fold cross-validation were used to optimize the C parameter. Class weights addressed data imbalance, and Youden’s J statistic determined the optimal prediction threshold. Model performance was assessed by AUC, Accuracy, Sensitivity, Specificity, and F1-score on the test set. The logistic model included three predictors: level of consciousness (OR=5.24, 95% CI 3.04–9.03), physical restraint (OR=3.76, 95% CI 1.80–7.84), and pre-ICU surgery (OR=2.74, 95% CI 1.19–6.31). The model achieved an AUC of 0.77 (5-fold CV: 0.81 ± 0.03) with an optimal cutoff of 0.67. Test performance showed accuracy 0.84, sensitivity 0.58, specificity 0.86, and F1-score 0.38, indicating good discrimination but limited sensitivity for high-risk patients. This study identified three risk factors for delirium in non-induced ICU patients. The developed model showed good performance, particularly in identifying low-risk patients. Although sample imbalance limited high-risk detection, the model can serve as a rapid clinical risk assessment tool. Future optimization is needed to improve high-risk patient recognition.

## Exploring the influences of digital job demands and resources on hospital nurses’ attachment to workplace and turnover intention

Yi-Wen Shih^1^, Hao-Yun Kao^1^

^1^Kaohsiung Medical University, Taiwan

Digital information and communication technology (DICT) have been widely adopted in healthcare workplaces and are often associated with improvements in efficiency and work processes. However, increasing system complexity and the speed of information exchange may also place additional demands on employees. Based on the Digital Job Demands–Resources model, this study offer how traditional and digital job demands and resources affect nurses’ stress, motivation, and organizational implications, including attachment to the workplace and turnover intentions. An online survey adapted from Scholze and Hecker (2024) was conducted using convenience sampling. Participants were nurses employed at four hospitals within a medical system in Taiwan. Data were collected between January and March 2026 and analyzed using Partial Least Squares Structural Equation Modeling (PLS-SEM). Digital job demands were positively associated with both stress and motivation, whereas traditional job demands showed no significant effects. Both traditional and digital job resources significantly enhanced motivation. Stress did not significantly predict organizational outcomes, while motivation demonstrated a strong positive effect. Most moderating effects were not supported. The model explained 30.5% of stress, 77.6% of motivation, and 48.4% of organizational outcomes. The study results suggest that digital job demands are more likely than traditional job demands to lead to higher stress among nurses, suggesting that digital job demands may represent a new source of strain, while employees appear to have adapted to existing workloads. In contrast, job resources consistently enhance work motivation across both traditional and digital contexts, and motivation plays a more important role than stress in explaining employee–organization relationships. In addition, most moderating effects were not supported, indicating that the positive effect of digital job resources on motivation remains relatively stable across different levels of job demands.

## Design and Validation of an LLM-Powered Intelligent Notification System for Occupational Injury Management

Hao-Yun Kao^1^, Wen-Ye Chang^2^, Chien-Ming Wu^3^, Chen-Cheng Yang^1^

^1^Kaohsiung Medical University, Taiwan

^2^National Taiwan University of Science and Technology, Taiwan

^3^Yuan’s General Hospital, Taiwan

The occupational injury reporting system serves as the cornerstone of national labor health surveillance; however, current practices are severely hindered by cumbersome administrative workflows and significant underreporting rates (ranging from 50% to 95%), which compromise data accuracy and policy formulation. To address these critical challenges, this study adopted the Design Science Research Methodology (DSRM) to develop an “Intelligent Notification System for Occupational Injury Management” integrated with Large Language Models (LLMs), deployed at a regional teaching hospital situated in a major industrial hub in Southern Taiwan. Transcending the limitations of traditional manual transcription, the system integrates a structured interview interface with the OpenAI API, utilizing Generative AI to automatically transform unstructured clinical narratives into standardized notification summaries. Furthermore, a rigorous “Human-in-the-loop” verification mechanism was implemented to ensure information integrity and mitigate risks associated with AI hallucinations. Empirical results from field testing demonstrate that the system significantly reduces administrative processing time by 60% to 70%, substantially alleviating the documentation burden on case managers. Additionally, the system achieved a score of 85 on the System Usability Scale (SUS), reflecting high user acceptance regarding its interface design and functional integration. This study confirms that integrating Generative AI into occupational injury management workflows not only optimizes administrative efficiency and data quality but also aligns with ESG sustainability goals through paperless operations, providing a concrete and scalable empirical model for the digital transformation of occupational health surveillance.

## Multi-Modal Radiomics for PET/CT Tumor Characterization

Jona Rose Llabres^1^, Johannes Gubat^2^, Ian Jasper Agulo^1^

^1^University of the Philippines Baguio, Philippines

^2^Department of Biomedical and Clinical Sciences, Linköping University, Sweden

This study develops a robust and quantitative framework for the precise characterization of tumor regions within PET/CT scans, a foundational step for accurately flagging abnormal scans and localizing malignancies in clinical oncology workflows. Utilizing a carefully curated cohort of 101 eligible patients from the Lung-PET-CT-Dx collection, sourced via The Cancer Imaging Archive (TCIA) [1], high-resolution CT and meticulously co-registered PET images were processed alongside their corresponding 3D lesion masks. A comprehensive suite of radiomic features was extracted using the PyRadiomics library [2], independently applied to both CT (density-based) and PET (metabolic activity) modalities. Rigorous standardization to a 1x1x1 mm pixel spacing was performed to ensure physical comparability across all volumetric data, adhering to the Image Biomarker Standardization Initiative (IBSI) guidelines [3]. In-depth statistical analysis focused on inter-modality feature relationships revealed that several critical shape-based radiomic features—specifically ‘Flatness’, ‘SurfaceVolumeRatio’, ‘MeshVolume’, ‘Sphericity’, and ‘Elongation’—demonstrated a perfect absolute correlation (1.0) between their CT and PET derivations. This correlation confirms the inherent stability and modality-independent nature of these morphological descriptors. Furthermore, distinct categories like First Order and Texture features exhibited significantly lower and broader inter-modality correlations, indicating their unique, non-redundant capture of distinct tumor biological and physical information [4]. This study provides a standardized multi-modal dataset and establishes a novel ‘physics database’ of radiomic features. The discovery of perfectly correlated shape features provides a powerful baseline for geometric stability, while the identified non-redundant features offer complementary insights. This foundational work is indispensable for the development of highly accurate, physics-driven predictive models crucial for advanced PET/CT scan abnormality detection and enhanced personalized cancer management.

## Evaluation of Lower Limb Muscle Function Using Surface Electromyography and Its Relationship with Handgrip Strength in Sarcopenia

Po-Hsiang Wang^1^, Ching-Fen Jiang^1^, Yu-Man Sun^1^

^1^I-Shou University, Taiwan

Sarcopenia is an age-related muscle degenerative syndrome associated with increased risks of falls, functional decline, and mortality in older adults. Handgrip strength (HGS) has been widely adopted as a core indicator of muscle strength in international consensus guidelines, such as the Asian Working Group for Sarcopenia (AWGS) 2019. However, HGS primarily reflects upper limb muscle strength, and its ability to represent lower limb muscle function remains controversial. This study aimed to investigate the relationship between HGS and lower limb maximal voluntary contraction (MVC) and to develop a surface electromyography (sEMG) system for objective assessment of lower limb muscle function in sarcopenia. A total of 70 participants were recruited and classified according to sarcopenia diagnostic criteria. Handgrip strength and MVC of major lower limb muscles were measured, and Pearson correlation analysis was performed to evaluate their association. In addition, a custom-designed multi-channel sEMG system was developed to synchronously record muscle activity from the quadriceps femoris, tibialis anterior, and gastrocnemius muscles in three physical performance tests. The results showed that HGS exhibited low correlation with lower limb MVC, suggesting that these measures are not highly consistent indicators of muscle strength. Furthermore, sEMG signal amplitude progressively decreased with increasing sarcopenia severity, reflecting reduced muscle activation.

## Improving AI-Driven Heart Failure Prediction: A Comparative Analysis of CNN and LSTM Architectures Using Holter-Derived SDNN Variability

Adnan Karaibrahimoglu^1^, Irfan Polat Kuzdere^1^, Nurgul Yasar^1^

^1^Suleyman Demirel University, Türkiye

Early detection of heart failure (HF) remains a critical clinical challenge, with autonomic dysfunction often preceding symptomatic decompensation. While traditional diagnostic methods are often reactive, Artificial Intelligence (AI) offers a proactive approach by identifying subtle patterns in continuous Holter monitoring data. This study evaluates the efficacy of two prominent deep learning architectures—Convolutional Neural Networks (CNNs) and Long Short-Term Memory (LSTMs)—for predicting HF using SDNN values. The analysis utilized 24-hour Holter monitoring data from 238 individuals, comprising both healthy controls and patients with varying stages of heart failure. The SDNN values were extracted to form a high-resolution time-series dataset. Two models were developed: a 1D-CNN designed to capture spatial/morphological features within the variability segments after Wavelet Transform and LU-Decomposition, and an LSTM network, a very recent type of Recurrent Neural Network (RNN) used in time series data, optimized to recognize long-term temporal dependencies and sequential fluctuations in the data. Performance was measured using accuracy, sensitivity, specificity, and F1-score, validated through 10-fold cross-validation. The comparative analysis revealed significant improvements in predictive accuracy when transitioning to deep learning models. The CNN architecture demonstrated weak feature extraction, achieving an accuracy of 58%. However, the LSTM model outperformed the CNN in recognizing the chronic, progressive nature of heart failure, achieving 65% accuracy and 77% sensitivity. The LSTM’s temporal gating mechanism proved more effective at distinguishing pathological SDNN depressions from transient physiological variations. Deep learning models significantly enhance the predictive value of Holter-derived SDNN data for heart failure. While CNNs are efficient for pattern recognition, LSTM networks provide a superior framework for analyzing the sequential nature of HRV. These findings suggest that integrating AI-driven LSTM models into wearable Holter technology could facilitate real-time, non-invasive risk stratification and early clinical intervention.

## Deploying AI-Enabled Learning Health Systems Under Real-World Constraints: Operational Lessons from Epilepsy, Dementia, and Bipolar Disorder

Dong-Gil Ko^1^

^1^University of Cincinnati, USA

Despite rapid progress in clinical artificial intelligence (AI), many AI systems fail to achieve sustained real-world impact – not because models underperform, but because learning is not embedded in workflows, governance, and operational decision-making. Learning Health Systems (LHS) address this challenge by integrating AI-enabled tools into continuous cycles of data capture, prioritization, intervention, and feedback that support organizational learning under real-world constraints. Using three LHS at different stages of maturity – epilepsy, memory/dementia, and bipolar disorder – I examine how AI-enabled learning loops are deployed and adapted across clinical contexts. Across all three domains, AI functions as assistive learning infrastructure rather than autonomous decision-making, helping surface actionable signals, focus scarce clinical attention, and support iterative learning. In epilepsy care, AI-supported risk stratification integrates longitudinal EHR data within Epic workflows to identify patients for targeted interventions. Standardized data capture and interventions increased seizure control from 33% to approximately 44% while improving documentation reliability. In memory and dementia care, digital cognitive assessment tools have been piloted in primary care but remain limited by adoption barriers. Current learning focuses on tracking diagnostic workup completion to identify workflow bottlenecks and redesign care pathways without increasing physician burden. Planned deployments illustrate the transition from experimentation to operational integration. In bipolar disorder care, efforts focus on automating patient-reported outcome collection through MyChart and exploring AI-supported risk stratification for metabolic monitoring among patients receiving antipsychotics. Across all three LHS, deployment success depends more on workflow alignment, clinician trust, and data reliability than model sophistication. AI creates greatest value when used to prioritize attention and structure learning rather than replace judgment. Heterogeneous maturity across conditions is not a failure mode but a defining feature of real-world LHS evolution. Together, these findings position AI-enabled LHS as reusable capabilities for deploying clinical AI at scale under real-world constraints.

## Predictive Analytics of Hospital Readmissions in Older Heart Failure Patients Using National Health Insurance Big Data

Yu-Jie Lin^1^, Hsin-I Kao^1^, Ying-Chun Li^1^

^1^National Sun Yat-sen University, Taiwan

Hospital readmissions among heart failure patients remain a major challenge for healthcare systems worldwide, particularly in aging populations. Early identification of high-risk patients is crucial to improving care quality, reducing healthcare costs, and enabling proactive disease management. As large-scale health databases become more accessible, health informatics and data-driven predictive analytics offer valuable opportunities to detect risk patterns and enhance clinical decision support. This study used predictive analytics on health data to evaluate hospital readmission risk among middle-aged and older patients with heart failure in Taiwan. Using the Taiwan National Health Insurance (NHI) database, we conducted a retrospective cohort study of patients aged 50 years or older with a heart failure diagnosis. Hospital readmissions were grouped by time after discharge: within 14 days, 30 days, 180 days, or more than 180 days. We included patient demographics, hospital characteristics, healthcare utilization measures, the Charlson Comorbidity Index (CCI), and medication use—including angiotensin-converting enzyme inhibitors (ACE inhibitors) and angiotensin receptor blockers (ARBs)—in predictive models. Multivariate logistic regression was used to identify key factors associated with hospital readmission risk. Among 2,173 hospitalized heart failure patients, 88.4% had at least one readmission. About 19.9% were readmitted within 14 days, 7.6% within 30 days, and 26% within 180 days. Patients with readmissions had significantly higher healthcare utilization, including inpatient and outpatient costs, medication expenses, and healthcare visits. Predictive modeling showed that advanced age, higher Charlson Comorbidity Index scores, and the use of ACE inhibitors or ARBs were significantly associated with increased risk of hospital readmission. These findings underscore the potential of health data analytics and predictive modeling to identify high-risk patients in large healthcare databases. Integrating predictive risk models into healthcare information systems can support clinical decision-making, improve disease management strategies, and reduce avoidable hospital readmissions among aging populations.

## Development of deep learning models for automatic detection of Pancreatic Cancer with Abdominal Ultrasound Images

Min-Kai Liao^1^, Hung-Wen Chiu^1^

^1^Taipei Medical University, Taiwan

Pancreatic cancer has a poor prognosis, and transabdominal ultrasound (TAUS) offers a non-invasive and accessible screening tool but has limited diagnostic accuracy. To develop a ResNet50-based deep learning model for automatic pancreatic cancer detection using abdominal ultrasound images. A total of 191 ultrasound images (105 pancreatic cancer and 86 normal pancreas) were collected and preprocessed. A ResNet50 convolutional neural network was trained to classify whole ultrasound images. Model performance was evaluated using accuracy, sensitivity, specificity, ROC curve, AUC, and loss curves.

The ResNet50 model achieved a validation accuracy of 91.67–100% after 30 training epochs. Training and validation accuracy remained stable with minimal overfitting, while loss curves demonstrated good convergence. These findings indicate that the proposed model provides accurate and robust classification performance and has promising potential for computer-aided detection of pancreatic cancer using abdominal ultrasound images.

## Controllable Diffusion Image Editing for Targeted Minority-Class Augmentation

Ping-Yen Hsieh^1^, Ting Ying Chien^1^

^1^Yuan Ze University, Taiwan

Chest radiography (CXR) and non-contrast head computed tomography (CT) are widely used in clinical diagnosis, but rare critical findings such as pneumothorax and intracranial hemorrhage (ICH) are often under-represented in real-world datasets. This class imbalance can bias classifiers toward majority classes and reduce sensitivity to clinically important minority cases. To address this problem, we propose a controllable diffusion-based image editing pipeline for targeted minority-class augmentation. The pipeline uses RadEdit-style counterfactual editing to insert or remove lesions while preserving patient-specific anatomy. Spatial control is provided by binary masks from ground truth annotations or curated mask banks, and semantic control is provided by structured prompts, including a severity scale from mild to severe. The generated samples are auditable and can be filtered before downstream training. We validate the pipeline in two settings: CXR pneumothorax using the SIIM-ACR segmentation dataset and non-contrast head CT ICH using 2D axial slices with weak labels. To evaluate controllability, we assess whether edits remain localized inside the target region, preserve non-edited anatomy, and follow monotonic severity changes. For downstream validation, synthetic samples are added only to the minority class with augmentation ratios of 0%, 5%, 10%, and 20%, while validation and test sets remain fully real. Models are evaluated using AUPRC, recall, and sensitivity at fixed specificity. Preliminary results suggest that controlled and quality-filtered synthesis can improve minority-class detection, whereas excessive or low-quality synthesis may reduce generalization. This study demonstrates the potential of controllable diffusion editing for imbalanced medical imaging across chest X-ray and brain CT tasks.

## Visual Finger Exercise Recognition System Integrated with Caregiving Robots for Delaying Cognitive Decline in the Elderly

Cheng-Yu Tsai^1^, Yi Lan^1^, Ting Ying Chien^1^

^1^Yuan Ze University, Taiwan

In response to the challenges of an increasingly aging society, the rising prevalence of dementia is placing a significant burden on social and healthcare systems. Academic research indicates that specific fine-motor finger exercises can effectively stimulate brain activity and delay cognitive decline. However, current long-term care systems face severe workforce shortages, making it difficult to provide consistent one-on-one guidance for these exercises. To address this, this study proposes a visual recognition system integrated into caregiving robots. To address this issue, this study proposes a visual recognition system that can be integrated into caregiving robots. In this application scenario, the caregiving robot guides the elderly in performing finger exercises and uses its built-in camera to capture their movements in real time and evaluate whether they are performed correctly. This significantly reduces the burden on caregivers and increases the elders’ engagement in rehabilitation. The system utilizes Python and the MediaPipe framework to detect and extract 21 3D hand landmarks (Figure 1) in real-time. These extracted features are then fed into a Support Vector Machine (SVM) model for action classification and recognition. To evaluate the system, we constructed a preliminary test set consisting of 186 gesture frames. Defining accuracy as the ratio of correctly classified frames to the total number of successfully extracted frames, our initial SVM model achieved an accuracy of 92.47%. These preliminary experimental results successfully verify the conceptual feasibility of automatically recognizing finger exercises using a camera and a machine learning model. Future work will focus on further training and optimizing the model with larger datasets to continuously improve recognition robustness, providing a concrete and effective automated solution for smart eldercare.

## Integrating Structural Cues and Multi-Level Representations for Generalizable Drug-Target Interaction Prediction

Hui Han^1^, Xiucai Ye^1^, Tetsuya Sakurai^1^

^1^University of Tsukuba, Japan

Drug-target interaction (DTI) prediction is a key component in modern drug discovery, supporting tasks such as drug repurposing and early-stage screening. While recent deep learning models have shown promising results, their performance often degrades when dealing with previously unseen drugs or targets. In addition, many existing approaches primarily focus on global representations, which may fail to capture critical local interaction patterns and structural characteristics of proteins. In this work, we introduce SMRDTI, a novel framework that combines structural information with multi-level representation learning for more robust DTI prediction. The model incorporates structure-derived protein features together with sequence-based drug representations, and models their interactions at different levels. A cross-attention mechanism is employed to explore detailed relationships between drug substructures and protein residues, particularly within predicted binding regions. Meanwhile, global representations are jointly optimized to capture broader contextual dependencies. Experimental results on multiple benchmark datasets demonstrate that the proposed method achieves consistent improvements over existing approaches under both standard and challenging evaluation settings, indicating its strong generalization capability.

## Learning Adaptive Sample Graphs for Multi-Omics Cancer Subtype Classification

Mengke Guo^1^, Xiucai Ye^1^, Tetsuya Sakurai^1^

^1^University of Tsukuba, Japan

Integrating multi-omics data is essential for capturing the complex heterogeneity of cancer and improving subtype classification. However, many existing approaches rely on predefined or heuristic graph structures and are limited in their ability to jointly model feature representations and sample relationships. In this study, we propose a novel graph-based framework for multi-omics cancer subtype classification that simultaneously performs feature learning and adaptive graph structure learning. Specifically, latent representations are first extracted from multiple omics datasets using convolutional autoencoders. To enhance biological relevance, survival-associated features are identified through Cox proportional hazards analysis. Based on these features, a variational graph autoencoder is employed to learn a probabilistic sample graph, allowing the model to capture uncertainty in sample relationships. The learned graph is then combined with feature representations and fed into a graph convolutional network for final classification. The proposed method is evaluated on eight cancer datasets spanning four omics types. Experimental results show that the model achieves stable and competitive performance compared with existing methods. In addition, downstream analyses, including survival prediction and gene-level investigation, demonstrate that the framework can provide interpretable insights and identify clinically relevant biomarkers. These results suggest that adaptive graph learning is a promising direction for improving multi-omics data integration in cancer research.

## Task-Aware Clustering Framework for Identifying Subtypes of Cognitive Decline

Weike Liu^1^, Xiucai Ye^1^, Tetsuya Sakurai^1^

^1^University of Tsukuba, Japan

Identifying hidden subtypes is important for understanding structural differences across different stages of cognitive decline. Existing studies have mainly relied on gray matter measures and neuropsychological assessments to examine differences among patient groups. However, such approaches may overlook the impact of white matter disruption in brain networks and therefore may not fully capture structural heterogeneity associated with cognitive decline. In this study, we applied a task-aware clustering framework that integrates two structural MRI modalities, gray matter and white matter, to identify latent structural subtypes. XGBoost models were trained on each modality to predict diagnostic labels, and SHAP values were extracted from each model to represent task-relevant feature contributions. These feature-contribution representations from gray matter and white matter were integrated using singular value decomposition (SVD), and clustered using spectral clustering to identify patient groups. The framework identified four clusters which showed different diagnostic compositions: two clusters were mostly HC, one was mainly MCI, and one included many participants with MCI and AD. The clusters also showed marked differences in gray matter and white matter features, as well as distinct patterns across multiple neuropsychological assessments. These findings support the utility of the task-aware framework for identifying latent subtypes beyond conventional diagnosis.

## Task-Aware Clustering of Gut Microbiome Data for Inflammatory Bowel Disease Stratification

Teruhito Matsunaga^1^, Xiucai Ye^1^, Tetsuya Sakurai^1^

^1^University of Tsukuba, Japan

Inflammatory bowel disease (IBD) shows substantial heterogeneity that is not fully captured by clinical indicators alone. Although the gut microbiome is closely associated with IBD, clustering raw microbial profiles does not always reveal disease-relevant variation. In this study, we explored a task-aware clustering framework for microbiome-based IBD stratification using the IBDMDB (HMP2) shotgun metagenomic dataset. An XGBoost classifier was trained to distinguish IBD from non-IBD samples, and SHAP-based feature contribution patterns were used to represent each sample. Clustering in this space identified groups with distinct distributions of clinically defined disease activity, suggesting that the framework captures clinically relevant heterogeneity beyond raw microbial composition alone. These findings support task-aware clustering as an interpretable approach to IBD stratification. Future work will examine species-level profiles and independent cohorts to further assess robustness and generalizability.

## Open-Source vs Proprietary GPT Models for Cancer Variant Interpretation: Benchmarking GPT-Oss 20B/120B Against GPT-4o on FoundationOne CDx

Kuan-Hsun Lin^1^, Tzu-Hang Kao^1^, Lei-Chi Wang^1^, Chen-Tsung Kuo^1^, Paul Chih-Hsueh Chen^1^, Yi-Chen Yeh^1^ and Yuan-ChiaChu^1^

^1^Taipei Veterans General Hospital, Taiwan

The interpretation of cancer genetic variants is essential for precision oncology, but the abundance of variants of unknown significance (VUS) in clinical sequencing reports remains a major challenge. Large language models (LLMs) offer a potential solution, yet most high-performing systems such as GPT-4o are proprietary and limited in transparency. In this study, we benchmarked open-source gpt-oss models (20b and 120b) on 10,506 variants from FoundationOne CDx and compared them against GPT-4o results from our previous work. The gpt-oss 120b model achieved the highest accuracy (0.77), surpassing GPT-4o (0.73) and gpt-oss 20b (0.71), with improved sensitivity for clinically actionable variants while maintaining high specificity for VUS. These findings highlight that open-source LLMs are rapidly approaching clinical applicability. Beyond oncology, their ability to support accurate and efficient interpretation of complex clinical data suggests broader applicability to other healthcare domains, including auditory medicine and sudden hearing loss.

## WebAR-Based Gamified Nutrition Platform for Dual-Purpose Health Intervention: System Design and Cross-Generational Validation

Han-chun Huang^1^, Hsu-Min Chang^1^, Yi-Fen Cheng^1^ and Tzu-Yin Wang^1^

^1^Tajen University, Taiwan

Augmented Reality (AR) has emerged as a promising technology for health interventions, yet deployment barriers—particularly application installation requirements—limit adoption in healthcare settings. This paper presents a WebAR-based gamified nutrition platform that addresses these accessibility challenges while serving dual health objectives across different user populations. The system architecture integrates AR.js and A-Frame frameworks to deliver browser-based AR experiences requiring no installation, with marker-based interaction using physical food cards representing Taiwan’s six major nutritional categories. A distinctive dual-purpose design enables the same platform to function as caregiver-assisted cognitive stimulation for elderly users with dementia and as self-directed gamified nutrition education for young adults. Cross-generational validation involving 12 elderly participants (aged 71–103 years, M = 82.8, SD = 9.6) from a long-term care community and 18 university students (14 female, 4 male) demonstrated comparable task performance across populations. Elderly participants achieved mean scores of 3.92 (SD = 1.00), with 66.7% attaining high performance (≥4 points), while students achieved mean scores of 4.28 (SD = 1.23), with 77.8% attaining high performance. These findings validate the universal accessibility of WebAR health interventions and demonstrate that appropriately designed AR platforms can effectively serve heterogeneous user populations through differentiated engagement mechanisms rather than differentiated technology.

## Enhancing Emergency Department Triage Workflow Efficiency via Speech Recognition and RAG: A System Design Based on the Taiwan Triage and Acuity Scale

Yu-Chien Sun^1^, Ting-Cheng Chu^1^, Yu-Jie Chou^1^, Kai-Ling Chu^1^, Audrey Pei-Chen Lin^1^, Tzu-Meng Yang^1^, Wei-Hong Shen^1^, Sin-Ping Li^2^, Chi-Jie Lu^1^

^1^Fu Jen Catholic University, Taiwan

^2^Fu Jen Catholic University Hospital, Taiwan

Emergency department (ED) overcrowding and nursing workforce shortages have fueled the demand for intelligent decision-support systems capable of enhancing triage efficiency and consistency. As the first point of clinical assessment, triage requires nurses to rapidly integrate patients’ chief complaints, symptoms, and vital signs under significant time pressure. However, chief complaints are often expressed as unstructured narratives, which poses challenges for information processing and standardized triage classification. This study proposes an intelligent ED triage decision-support system that integrates speech recognition, retrieval-augmented generation (RAG), and a locally deployed large language model (LLM) based on the Taiwan Triage and Acuity Scale (TTAS). The system comprises a React frontend and a Python FastAPI backend, with the LLM deployed on-premises via Ollama to address privacy and data-security concerns. Speech recognition is employed to convert nurse-patient conversations into text, while a TTAS-oriented RAG knowledge base is utilized to retrieve guideline-based evidence. The LLM then generates structured chief-complaint summaries, symptom recommendations, and triage-level suggestions grounded in the retrieved clinical knowledge. The proposed framework aims to reduce the documentation burden, improve information structuring, enhance triage consistency, and provide traceable, guideline-based recommendations while preserving clinician oversight. By combining local LLM deployment with RAG-driven decision support, the system offers a practical, privacy-preserving approach to intelligent triage assistance in real-world emergency care environments.

## Ensemble Learning-Driven Cyber Attack Detection in IoHT under a Zero Trust Architecture: A Primary Study

Tian-Fu Lee^1^, I-Pin Chang^2^, Tsung-Hung Lin^3^

^1^Tzu Chi University, Taiwan

^2^National Taiwan University of Science and Technology, Taiwan

^3^National Chin-Yi University of Technology, Taiwan

As the Internet of Health Things (IoHT) progressively interlinks medical apparatus with healthcare information technology systems, the susceptibility to cyber threats, including ransomware, denial-of-service attacks, and data infringements, intensifies, thereby jeopardizing sensitive patient information. To devise a resilient and adaptive defense mechanism aimed at safeguarding IoHT environments against the escalating frequency and complexity of cyber-assaults. This research introduces an ensemble learning-based cyber-attack detection framework assessed on the ECU-IoHT dataset. The framework incorporates a dynamic risk scoring algorithm within a Zero Trust Architecture (ZTA). Rather than employing a conventional binary classification of “attack or no attack,” it leverages continuous verification to produce an ongoing risk score, facilitating the automatic modification of security protocols. The ensemble-based ZTA model markedly surpasses preceding methodologies. In particular, the proposed Voting Classifier attained an extraordinary accuracy of 99.10%, exceeding the performance of foundational models such as Logistic Regression and Random Forest. It also exhibited high precision (0.9909), recall (0.9910), and an F1-score of 0.9905, reflecting outstanding predictive reliability. The amalgamation of model aggregation (ensemble learning) with a dynamic Zero Trust Architecture results in enhanced robustness. By autonomously adjusting its security thresholds in response to evolving attack patterns, the proposed system offers a highly efficient and reactive defense mechanism specifically designed for IoHT environments.

## Integration of Electronic Medical Record Database Security and Artificial Intelligence on Gastric Cancer Recognition

Tian-Fu Lee^1^, Jyun-Guo Wang^1^, Jui-Sheng Hung^2^, I-Pin Chang^3^

^1^Tzi Chi University, Taiwan

^2^Hualien Tzu Chi Hospital, Taiwan

^3^National Taiwan University of Science and Technology, Taiwan

Gastric cancer is one of the most common cancers worldwide. Its morphology under a microscope is diverse, easily misdiagnosed as non-cancerous inflammation. Therefore, this study developed an artificial intelligence (AI)-based model to assist pathologists and physicians in detecting cancerous areas using unlabeled digital endoscopic images. Furthermore, while electronic medical records offer convenience and portability, many database systems have not adequately considered information security, leading to frequent security vulnerabilities. Therefore, in addition to utilizing AI models to improve the efficiency and accuracy of gastric cancer region labeling detection, this study also fully considered information security and patient privacy issues related to image-based medical records during implementation.

**Table:** 

**Author Index**
A
Ahmad Gul, Azami, 4
Audrey Pei-Chen, Lin, 16
Adnan, Karaibrahimoglu, 10
B
Ben, Harris-Roxas, 2
C
Chieh-Kuan, Hsu, 8
Chen-Tsung, Kuo, 15
Chih-Han, Leng, 3
Chi-Jie, Lu, 16
Cheng-Yu, Tsai, 13
Chien-Ming, Wu, 9
Chen-Cheng, Yang, 9
D
Dong-Gil, Ko, 11
Dipankar, Srirag, 2
G
Grace, Yao, 3
H
Hung-Wen, Chiu, 12
Hsiao-Wen, Chuang, 4
Hsu-Min, Chang, 15
Han-chun, Huang, 15
Hui, Han, 13
Hao-Yun, Kao, 9
Hsin-I, Kao, 12
Hsin-Ching, Yang, 5
I
Ian Jasper, Agulo, 9
I-Pin, Chang, 16, 17	
Irwanto, Irwanto, 6
Irfan Polat, Kuzdere, 10
Irwin, Redlener, 5
J
Johannes, Gubat, 9
Jia-Chi, Hsu, 3
Jui-Sheng, Hung, 17
Jarone, Lee, 5
Jona Rose, Llabres, 9
Jyun-Guo, Wang, 17
Jay, Wu, 3
Joseph, Zhu, 2
K
Kai-Ling, Chu, 16
Kuan-Hsun, Lin, 15
Karen, Redlener, 5
L
Li-Chung, Lin, 3
Lei-Chi, Wang, 15
M
Mary, Showstark, 5
Min-Kai, Liao, 12
N
Nazanin, Ghahreman-Falconer, 2
Nur, Iriawan, 6
Nataly, Martini, 2
Noppadon, Tangpukdee, 2
Nurgul, Yasar, 10
O
Olha, Maltseva, 5
Oleksii, Vlasov, 5
P
Paul Chih-Hsueh, Chen, 15
Ping-Yen, Hsieh, 12
Padmanesan, Narasimhan, 2
Po-Hsiang, Wang, 10
Pei-Jane, Wu, 5
Polrat, Wilairatana, 4
Q
Quoc, Dung Nguyen, 2
R
Robert, Arnot, 5
Ratikorn, Boonchai, 6
Roberto, Forero, 2
S
Shinn-Ying, Ho, 7
Shabrina, Imanina, 6
Sin-Ping, Li, 16
Sant, Maungnoicharoen, 4
Sreyon, Murthi, 2
Stella, Nam, 5
Shane, Scahill, 2
T
Ting-Cheng, Chu, 16
Ting Ying, Chien, 12, 13
Taufiq, Hidayat, 6
Tzu-Hang, Kao, 15
Tian-Fu, Lee, 16, 17
Tsung-Hung, Lin, 16
Tetsuya, Sakurai, 13
Tzu-Meng, Yang, 16
W
Wen-Ye, Chang, 9
Wei-An, Ke, 7
Wei-Hong, Shen, 16
X
Xiucai, Ye, 13
Y
Yuan-Chia, Chu, 15
Yi-Fen, Cheng, 15
Yu-Jie, Chou, 16
Yi-Chen, Huang, 4
Yu-Lu, Huang, 8
Yuliia, Kardash, 5
Yi, Lan, 13
Ying-Chun, Li, 5, 12
Yen-Tze, Liu, 7
Yu-Jie, Lin, 12
Yi-Wen, Shih, 9
Yu-Tai, Shih, 3
Yu-Chien, Sun, 16
Tzu-Yin, Wang, 15
Yi-Chen, Yeh, 15

